# Alternative strategies for *Chlamydia* treatment: Promising non-antibiotic approaches

**DOI:** 10.3389/fmicb.2022.987662

**Published:** 2022-11-23

**Authors:** Chen Hou, Yingqi Jin, Hua Wu, Pengyi Li, Longyun Liu, Kang Zheng, Chuan Wang

**Affiliations:** ^1^School of Basic Medicine, Hengyang Medical College, Institute of Pathogenic Biology, University of South China, Hengyang, China; ^2^Department of Clinical Laboratory, Affiliated Hengyang Hospital of Southern Medical University, Hengyang Central Hospital, Hengyang, China

**Keywords:** *Chlamydia*, chlamydial infection, antibiotic therapy, anti-chlamydial compounds, non-antibiotic approaches

## Abstract

*Chlamydia* is an obligate intracellular bacterium where most species are pathogenic and infectious, causing various infectious diseases and complications in humans and animals. Antibiotics are often recommended for the clinical treatment of chlamydial infections. However, extensive research has shown that antibiotics may not be sufficient to eliminate or inhibit infection entirely and have some potential risks, including antibiotic resistance. The impact of chlamydial infection and antibiotic misuse should not be underestimated in public health. This study explores the possibility of new therapeutic techniques, including a review of recent studies on preventing and suppressing chlamydial infection by non-antibiotic compounds.

## Introduction

### *Chlamydia* and the epidemiology of chlamydial infection

*Chlamydia* is an obligate intracellular, multi-host, and gram-negative pathogen with a unique developmental cycle (Elwell et al., 2016; Zhong, 2017). It has two main, common, morphologically distinct forms: the infectious elementary body (EB) and the reproductive reticulate body (RB) (Cossé et al., 2018). Elementary bodies can transition into intermediate bodies (IBs) and later differentiate into RBs in an inclusion process (Núñez-Otero et al., 2021). It is worth noting that if exposed to stressful conditions, like penicillin, IFN-γ, or lack of essential nutrients *in vitro, Chlamydia* may enter a stable state containing enlarged but non-infectious aberrant RBs (ABs) (Hammerschlag, 2002; Panzetta et al., 2018). Persistence represents the attempt of the host to control *Chlamydia*. Meanwhile, *Chlamydia* has developed corresponding mechanisms for escaping the host immune response, especially by constructing an immune-evasive persistent state. The persistent state is helpful for *Chlamydia* since it is a widespread pathogen (Gracey and Inman, 2011).

Most chlamydial species are pathogenic and infectious, particularly *Chlamydia trachomatis, Chlamydia pneumonia*, and *Chlamydia psittaci*, which are human pathogens. In 2020, the Centers for Disease Control and Prevention (CDC) reported that the number of *C. trachomatis* infections had reached 1,579,885 cases in the United States (Sexually Transmitted Disease Surveillance, 2020). This pathogen is one of the major causes of sexually transmitted infections (STIs) in the United States. It can cause anogenital tract infectious diseases and multiple sequelae, including pelvic inflammatory disease, ectopic pregnancy, infertility, and epididymitis. It can also accelerate the acquisition and transmission of the human immunodeficiency virus (HIV) in both sexes (Cornelisse et al., 2017; Panzetta et al., 2018; National Academies of Sciences, Engineering, and Medicine et al., 2021). As the most common infections among humans, STIs have caused significant morbidity and mortality in the United States and worldwide. *C. trachomatis* infections can also lead to conjunctivitis, trachoma, and subacute and afebrile pneumonia. Conversely, *C. pneumoniae* is the primary cause of human respiratory diseases, including pneumonia and bronchitis. Approximately 10% of community-acquired pneumonia and 5% of bronchitis result from *C. pneumoniae* infection (Roulis et al., 2013). As knowledge of *C. pneumoniae* increases, it appears to be associated with certain chronic diseases, including asthma, chronic obstructive pulmonary disease, atherosclerotic cardiovascular disease, and lung cancer (Porritt and Crother, 2016; Crother et al., 2019; Khoshbayan et al., 2021; Premachandra and Jayaweera, 2022). However, it is crucial to determine the exact relationship between *C. pneumoniae* infection and related diseases through further clinical studies. Psittacosis caused by *C. psittaci* is a zoonotic disease with various clinical symptoms, such as fever, headache, muscle aches, malaise, chills, pneumonia, non-productive coughing, and respiratory distress (Beeckman and Vanrompay, 2009; Shaw et al., 2019; Li N. et al., 2021). A previous study found significant differences in the epidemiology of psittacosis by gender by descriptively analyzing psittacosis cases reported in Japan from 2007 to 2016. Yet, the reasons leading to gender differences are uncertain and remain to be solved (Kozuki et al., 2020). Overall, *Chlamydia* can infect various areas, including the ocular mucosa, respiratory tract, and anogenital tract, causing a variety of infectious diseases and complications in humans and animals. In terms of public health, the chlamydial infection has long been an adversary not to be underestimated. Thus, safe and effective treatment should be provided for patients with chlamydial infections.

### Traditional therapy: Antibiotics

Antibiotics are often recommended for clinical chlamydial infections (Schachter and Caldwell, 1980; Clarke, 2011). Notably, there can be differences in the clinical symptoms of infection, sensitivity to antibiotics, and caution against various antibiotics among different infection sites. Thus, it is necessary to choose a suitable treatment based on the chlamydial infection site (Doernberg et al., 2020; Man et al., 2021). According to the STI treatment guidelines presented by the CDC in 2021 (CDC, 2022), doxycycline, azithromycin, levofloxacin, amoxicillin, erythromycin base, or ethylsuccinate are used to treat *C. trachomatis* infection. However, particular recommendations and different regimens should be followed for *C. trachomatis* infections in pregnant women, neonates, infants, children, adolescents, and adults.

Although antibiotics have been considered the standard treatment for chlamydial infections, some disadvantages of the treatment make it somewhat limited. Misuse of antibiotics is likely to disrupt the gut microbial community and increase the risk of the emergence of antibiotic-resistant chlamydial species or bacteria (Fröhlich et al., 2016; Angelucci et al., 2019; Benamri et al., 2021). For example, treating *C. trachomatis* infections with azithromycin can lead to resistance in *Streptococcus pneumoniae* and *Mycoplasma genitalium* (Jensen et al., 2008; O'Brien et al., 2019; Núñez-Otero et al., 2021). Many tetracyclines, i.e., antibiotic growth promoters, are supplied with livestock feed and may be the main reason for inducing stable tetracycline resistance in *Chlamydia suis* (Roberts, 1996; Chopra and Roberts, 2001; Dugan et al., 2004). Presently, human chlamydial strains do not show tetracycline resistance. Although antimicrobial resistance in *Chlamydia* is currently sporadic in the clinical setting, it still poses a public health threat (Dugan et al., 2004). Particularly, tetracyclines (i.e., doxycycline) are used as the first-line treatment for *C. trachomatis* at all infection sites (except Trachoma) and increase this misuse (Lau et al., 2021; Fairley et al., 2022). It is worth noting that various gene mutations in Chlamydial species are associated with antibiotic resistance. For example, *C. trachomatis* and *C. psittai* may develop resistance to macrolides through mutations in the 23S rRNA gene.

Furthermore, gene sequencing of the susceptible and resistant *C. trachomatis* strains revealed mutations in the A2057G, A2059G, and T2611C peptidyl transferase regions of the 23S rRNA gene related to antibiotic resistance (Benamri et al., 2021). In addition, antibiotic misuse is closely associated with treatment failure of chlamydial infection (Kardas et al., 2005). Research has shown that heterotypic resistance and single-dose therapy with a bacteriostatic antibiotic may be a biologically rational explanation for the failure of azithromycin treatment of *C. trachomatis* (Horner, 2012). *In vitro* evidence showed that if *Chlamydia* is exposed to stress conditions caused by penicillin (belonging to β-lactam antibiotics) and IFN-γ during replication, it may enter a particular state called “chlamydial persistence” (Hocking et al., 2015; Panzetta et al., 2018). Stress conditions mainly include impaired ATP production, oxidative stress, feedback regulation of cellular core processes, induction of the stringent response with the alarmone guanosine tetra- and pentaphosphate or the RpoS-mediated general stress response, and the added release of the toxin component (Eisenreich et al., 2022). Persistent chlamydial infection, a health hazard that should not be ignored, usually has a long incubation period and shows mild or even asymptomatic clinical symptoms. Also, most *C. trachomatis* infections, especially genital, rectal, and oral infections, are asymptomatic (Bogdanov et al., 2014; Vodstrcil et al., 2015; Adamson and Klausner, 2018; Hiransuthikul et al., 2019; Durukan et al., 2020). Therefore, it is essential to screen for STIs to prevent and control *C. trachomatis* infections and maintain public health safety. Currently, the most common approach for detecting *C. trachomatis* is nucleic acid amplification tests (NAATs), which have a high degree of sensitivity and specificity (Gaydos et al., 2004; Durukan et al., 2020).

## Non-antibiotic approaches

Chlamydial infection and antibiotic resistance are important threats to public health safety. It is crucial to optimize the use of antibiotics and develop new drugs or treatments that selectively target *Chlamydia* to limit the likelihood of the emergence of resistant strains. Several researchers have recently suggested that certain non-antibiotic substances can inhibit chlamydial infection through various mechanisms and may be promising candidates for anti-Chlamydial drugs (Table 1).

**Table 1:** Past studies exploring the anti-chlamydial properties of non-antibiotic approaches.

**Category**	**Designation**	**Chlamydial specie**	**Model**	**Antibacterial mechanism**	Shortcomings^*^
Synthetic Drugs	Broad-Spectrum Antiviral Compound ST-669	*C. trachomatis;* *C. pneumoniae*	Vero cells; HeLa cells; McCoy cells	•Affecting intracellular growth in a host-cell-dependent manner; •Interrupting the normal development of chlamydial inclusions through lipid droplet-dependent processes	•The efficacy is hard to predict
	Designated compound 1 (C1); A small-molecule inhibitor of type III secretion INP0400	*C. trachomatis*	HeLa 229 epithelial cells; McCoy cells; Mouse vaginal infection model	•Inhibiting RB to EB differentiation; •Inhibiting the type III secretion system of *Chlamydia*	•The efficacy and pharmacokinetics properties are hard to predict
	Lipopolysaccharide-Binding Alkylpolyamine DS-96	*C. trachomatis*	HeLa 229 cells	•This effect can be manifested at an early stage; •Inhibition of EB attachment and entry by binding to lipooligosaccharide (LOS)	•Has cell toxicity, and toxicity studies need to be further investigated
	Synthetic polymers PSS and SPS	*C. trachomatis;* *C. muridarum*	HeLa 229 cells Female outbred Swiss Webster mice	•Effectively block the chlamydial attachment and/or reduce host-pathogen interactions; •The antichlamydial effect of these drugs performed in a concentration-dependent manner	•No sigmoid curve or absolute infection prophylaxis was observed with SPGG at any increase
Polyphenols	Baicalin Luteolin Catechins **n**	*C. trachomatis* *C. pneumoniae* *C. pneumoniae*	Hep-2 cells; C57BL/6 mice C57BL/6J mice HeLa 229 cells	•Inhibiting phosphorylation cascades; •Reducing the production of chemokines; •Damage the plasma membrane by destroying the permeability of the lipid bilayer	•High concentrations of this class of compounds are cytotoxic
**Category**	**Designation**	**Chlamydial specie**	**Model**	**Targeting aspects of chlamydial infection**	**Shortcomings** ^*^
Lipidic	Five active synthetic lipids; 3-O-octyl-sn-glycerol [3-OG]; Fatty Acids and Monoglycerides	*C. trachomatis*	McCoy cells; Mouse fibroblast cells; McCoy mouse fibroblast cells; McCoy cells	•Destroying the membrane of the pathogen to inhibit the infection	•High concentrations of this class of compounds are cytotoxic
Peptides	Transferrin; WLBU2 Peptide; Cecrotin peptides; Cathelicidin peptides; Spider venom peptides; Antimicrobial Peptide Melittin	*C. psittaci;* *C. trachomatis;* 25 *Chlamydia* strains	HD11 cells; McCoy cells; Turkeys; McCoy mouse fibroblast cells; LLC-MK2 cells; Human red blood cells; HeLa cells; HEK293 cells	•Interfering with the *Chlamydia* adhesion to cells through decreasing transmembrane potential of host cells	•The therapeutic effect is slow, slower than conventional antibiotic therapy
Immune substances – cytokines	Interferon-γ (IFN-γ); Tumor Necrosis Factor (TNF); Interleukin (IL-4, IL-1α, IL-10, IL-23)	*C. muridarum*; *C. trachomatis;* *C. pneumoniae*	BALB/c mice; C57BL/6 mice; TNFR1 mice; C57BL/6 mice; TNFR1 mice; C57BL/6 mice	•Enhancing host immune response; •Infection can be directly restricted by inducing tryptophan catabolism and nitric oxide	•The pleiotropy of cytokine receptors and undesired activation of off-target cells
Vaccines	Subunit peptide vaccine; Recombinant vaccine; Mucosal vaccine^**^	*C. muridarum* *C. trachomatis*	Female BALB/c mice; C57BL/6 mice; C57BL/6 and BALB/c mice	•Preventing early infection •Shortening the duration of infection	•Causing abnormal host immune response

* All therapies still are preclinical.

** In Clinical trial 1 phase as a prophylactic vaccine.

### Synthetic drugs

Antibiotic resistance is a major public health concern; hence, compounds that are selectively effective against *Chlamydia* are of great interest for reducing pressure on antibiotic resistance in commensal and pathogenic bacteria. Here, we list several synthetic compounds with potential anti-Chlamydial activity and discuss their corresponding mechanisms.

Wolf et al. demonstrated that a small molecule of Yersinia T3SS inhibitor, designated compound 1 (C1), inhibits the development of *C. trachomatis* (Wolf et al., 2006). The expression of T3SS presumably helps *Chlamydia* establish and maintain the intracellular infection status by secreting anti-host proteins. Thus, inhibiting the T3SS compound is promising for treating chlamydial infection (Wolf et al., 2006). This anti-Chlamydial mechanism has also been confirmed in subsequent studies. For example, Muschiol et al. demonstrated that INP0400 (a small-molecule inhibitor of T3SS) also played a distinctive role in different stages of *C. trachomatis* infection (Muschiol et al., 2006). At a concentration of 10 μM, INP0400 can inhibit RB replication and reduce the number of inclusions in the early stage (Muschiol et al., 2006). At the later stage, INP0400 can cause the separation of RBs from the inclusion membrane and inhibit the transformation of RB into EB, leading to a significant decrease in infection (Muschiol et al., 2006). Another study found that inhibitors of bacterial type III secretion (T3S), ME0177 and ME0192, may be considered for systemic and topical treatment of chlamydial infection by individual pharmacokinetic analysis (Ur-Rehman et al., 2012). Importantly, ME0192 can inhibit *C. trachomatis* genital infection in mice but not the normal vaginal flora (Ur-Rehman et al., 2012). Results from this study suggest that vaginal microbicides may be considered candidates against local chlamydial infection. In particular, the nonoxynol-9 (N-9) formulated foam was demonstrated to prevent simian immunodeficiency virus (SIV) and simian-human immunodeficiency virus (SHIV) infection in rhesus macaques (Miller et al., 1992; Weber et al., 2001). However, some clinical trials suggested that N-9 does not protect against certain STI-induced microbes, including *Neisseria gonorrhoeae, C. trachomatis, Trichomonas vaginalis*, and HIV (Wilkinson et al., 2002).

Moreover, gonorrhea and the HIV infection rate appeared to be higher in women using the N-9 gel (Richardson et al., 2001; Van Damme et al., 2002). Nonoxynol-9 was not developed into a vaginal microbicide mainly due to its low efficiency in killing microbes and causing vaginal epithelium damage that promotes microbes' entry into women's bodies. Thus, the integrity of the female reproductive tract is considered to be an important evaluation criterion for vaginal microbicides (Tanphaichitr et al., 2016). Detection of vaginal toxicity, such as irritation, ulcerations, and histological inflammation of the vaginal microbicides, also plays a vital role.

Additionally, Osaka and Hefty (2014) found that low concentrations of lipopolysaccharide-binding alkyl polyamine DS-96 can block EB infection during the attachment phase and inhibit the growth of *Chlamydia*. Synthetic polymers, like sulfonated synthetic polymers called poly (sodium 4-styrene sulfonate/acid; PSS) and polyanetholsulfonic acid sodium salt (SPS), can suppress the formation of chlamydial inclusion in a concentration-dependent manner (Gallegos et al., 2018). Several small-molecule inhibitors with anti-Chlamydial activity have been reported. For instance, adding the inhibitor JO146, which targets the HtrA serine protease, during the replication phase of *C. trachomatis* can destroy the typical morphology of RB, decrease the inclusion size, and finally inhibit the formation of viable EB (Gloeckl et al., 2013). Similarly, the small-molecule inhibitor H89 decreases the production of chlamydial progeny by reducing RB replication and interfering with RB to EB conversion (Muñoz et al., 2021). In addition, a molecule inhibitor, MK2206, can alter host lipid synthesis and cholesterol transfer to reduce the conversion of RB to EB (Muñoz et al., 2022). Based on these studies, small-molecule inhibitors interfere with the development and infection of *Chlamydia* by reducing the production of EB, altering inclusion size, and disrupting RB to EB conversion. Inhibitors targeting the developmental cycle of *Chlamydia* may be a new anti-Chlamydial therapeutic strategy.

### Natural compounds and anti-infective action

#### Polyphenols

Polyphenols are bioactive molecules widely distributed in fruits, vegetables, grains, and beverages. Some have the potential for an antibacterial activity where antibiotic synergy inhibits bacterial virulence (Vuorelaa et al., 2004; Cushnie and Lamb, 2011; Fiorentini et al., 2015). Thus, studies have tried to use polyphenols in anti-Chlamydia experiments and found that certain compounds have specific anti-Chlamydia effectiveness (Table 1). Polyphenols have multiple modes of action against *Chlamydia*, but the exact mechanism of action needs further study. Alvesalo et al. showed that polyphenols' structure might influence the anti-Chlamydial effect (Alvesalo et al., 2006). Natural flavonoids and synthetic structural analogs have been shown to inhibit *C. pneumoniae* in *in vitro* experiments, and flavonoids without sugar moieties have higher anti-*C. pneumoniae* activity than those with other structures (Alvesalo et al., 2006).

Catechins, a type of flavan-3-ol flavonoid, are ubiquitous constituents of vascular plants and have broad-spectrum antimicrobial properties (Lambert et al., 2007; Sajilata et al., 2008). It can damage the plasma membrane by disrupting the lipid bilayer's permeability (Lambert et al., 2007). This direct antibacterial mechanism supports the broad-spectrum antibacterial properties observed in other studies (Lambert et al., 2007; Sajilata et al., 2008; Li et al., 2019). Catechins can control influenza viruses, coronaviruses, and oral microbial infectious diseases (Lambert et al., 2007; Furushima et al., 2018; Reygaert, 2018; Li et al., 2019; Yang et al., 2021). Yamazaki et al. investigated the anti-Chlamydial effects *in vitro* of five catechin-rich tea polyphenols, including catechin, epicatechin, epigallocatechin, epicatechin gallate, and epigallocatechin gallate (Yamazaki et al., 2003, 2005). All the tea polyphenols tested had inhibitory effects on chlamydial proliferation. The *C. pneumoniae* strains, AC-43 and AR-39, were inhibited entirely with the tea polyphenols at concentrations of 1.6 and 0.8 mg/mL, respectively. For serotypes D and L2, *C. trachomatis* was completely suppressed with the tea polyphenols at concentrations of 1.6 and 0.4 mg/mL, respectively.

Moreover, epigallocatechin gallate is considered to be the main component of the observed antibacterial effect (Yamazaki et al., 2003, 2005). Studies have shown that each tea polyphenol can be applied topically but not orally to treat systemic infections. Because the concentration of tea polyphenols required for complete inhibition of *C. trachomatis* is high compared to antibiotics, the toxicity will increase with an increased dose; thus, tea polyphenols are not currently suitable for treating systemic infections (Yamazaki et al., 2003, 2005). More research is needed to determine if targeting the structure of the tea polyphenol can lead to the creation of more potent systemic drugs.

Luteolin is a flavone found in vegetables, fruits, and medicinal herbs (Aziz et al., 2018). It can inhibit phosphorylation, a proinflammatory cytokine, and chemokine production *in vitro* or in animal models, and it has antioxidant, antibacterial, and anti-inflammatory properties (Kotanidou et al., 2002; Imran et al., 2019). Tormakangas et al. have evaluated the effects of acute *C. pneumoniae* infection treatment with the flavonoids, quercetin, luteolin, alkyl gallate, and octyl gallate in a mouse model and found that luteolin was able to suppress *C. pneumoniae* inflammation in lung tissue. Their study suggests that luteolin attenuates the inflammatory response induced by chlamydial infection through a cascade of NF-κB-mediated effects. Luteolin may also interfere with the mitochondrial pathway to induce apoptosis and eliminate the anti-apoptotic effect of *Chlamydia* (Törmäkangas et al., 2005). However, it is essential to note that luteolin treatment can reduce the production of *C. pneumoniae*-specific antibodies, possibly because luteolin directly reduces the natural inflammatory process and decreases the immune response (Törmäkangas et al., 2005).

Baicalin is a flavone derived from the raw, dry root of Scutellaria baicalensis, which has anti-inflammatory, anti-tumor, and antiviral activity (Jiang et al., 2020). Baicalin is an effective anti-Chlamydial agent and can potentially treat anti-Chlamydial infectious diseases both in cells and in animal experiments (Hao et al., 2010). A previous study has shown that baicalin can successfully block the *C. trachomatis* infection of HEP-2 cells (Hao et al., 2009). Ongoing research demonstrates that baicalin might affect the expression of chlamydial protease-like activity factor (CPAF) in HEP-2 cells with *C. trachomatis* infection. Baicalin can target and down-regulate CPAF production so that the immune system can detect chlamydial infection more effectively (Fan et al., 2002; Hao et al., 2009). Furthermore, studies have demonstrated that CPAF degrades host transcription factors, including RFX5, which is necessary for *Chlamydia* to evade host immune recognition defense mechanisms (Hao et al., 2010). Hao et al. have suggested that baicalin can block *C. trachomatis* infection by inhibiting the toll-like receptors 2 and 4 (TLR 2/4) and nuclear factor-κB (NF-κB) signaling pathways in genital tract cervical tissue infected with *C. trachomatis* in mice (Hao et al., 2012).

#### Lipids

Since some lipids have broad-spectrum antibacterial effects, especially targeting gram-negative bacteria (Lee et al., 2013), it is theorized that lipids may be used against *Chlamydia* (gram-negative bacterium). In fact, experiments have shown that parts of lipids have anti-chlamydia activity and can destroy the membrane, which may be the primary mechanism (Yoon et al., 2015, 2018; Casillas-Vargas et al., 2021). Bergsson et al. have proved that monocaprine, lauric acid, and decanoic acid have the strongest activity against *C. trachomatis* infection among 12 lipidic compounds (Bergsson et al., 1998). Synthetic lipids have also been shown to have potential as topical fungicides (Mansouri et al., 2021). Lampe et al. studied five active synthetic lipids developed from human milk (Lampe et al., 1998). When applied at 7.5 mM for 120 min, 2-O-octyl-sn-glycerol completely inhibited the growth of *C. trachomatis* compared with the other four lipids (Lampe et al., 1998). Considering that *C. trachomatis* is a sexually transmitted disease, the study also evaluated the anti-Chlamydial effect of 2-O-octyl-sn-glycerol under conditions similar to the human vagina (10% of human blood, pH 4.0–8.0). After exposing EBs to 50 mM 1-O-hexyl-sn-glycerol for 90 min, EBs appeared to have a hollow shell, ruptured cell membranes, and cytoplasmic contents leaking from the cell (Lampe et al., 1998). These results support the direct damage and/or destruction of the chlamydial lipid membrane and the potential anti-Chlamydial activity of 2-O-octyl-sn-glycerol (Lampe et al., 1998). Furthermore, Skinner et al. explored the development of topical microbicides using the synthetic lipid 3-O-octyl-sn-glycerol [3-OG] and the engineered antimicrobial peptide, WLBU2, as active compounds (Skinner et al., 2010). The authors found that both WLBU2 and 3-OG were effective against *C. trachomatis in vitro*, and their synergistic inhibitory activities were considerably enhanced (Skinner et al., 2010). Existing studies have demonstrated that lipids have anti-Chlamydial properties mainly related to destroying the pathogen's cell membranes. Still, the exact mechanisms and the selective effect against chlamydia must be explored.

#### Peptides

Some peptides consist of short amino acid chains that are common antibacterial protein compounds (Yasin et al., 2004). Lazarev et al. explored the use of the antimicrobial peptide melittin in treating chlamydia infections, the main active ingredient in bee venom, in the mid-1990s (Lazarev et al., 2002, 2005, 2007). *C. trachomatis* was inhibited *in vitro* by introducing and activating a recombinant plasmid vector expressing the melittin gene. Melittin not only has a direct bactericidal effect on cells (Lazarev et al., 2002) but can also restrict the adhesion of *Chlamydia* to cells by reducing the transmembrane potential of cells (Lazarev et al., 2005). Cathelicidin peptides, composed of amino acids (usually fewer than 50 amino acids) and cationic, are the building blocks of immune molecules with a wide range of antimicrobial or anti-Chlamydial activity (Francesco et al., 2013; He et al., 2018; Rowe-Magnus et al., 2019). Previous literature reported that SMAP-29 was the most potent antimicrobial peptide against various *Chlamydia* species compared with the five other antimicrobial peptides. Additionally, BMAP-27, BMAP-28, Bac7(1–35), and PG-1 have also been shown to reduce *C. trachomatis* and *C. pneumoniae* inclusion at a concentration of 10 μg/mL (Francesco et al., 2013). A follow-up study has shown that most swine *Chlamydia* isolates were sensitive to the same type of antimicrobial peptides, especially BMAP-29 (Donati et al., 2007).

Cecropins are a group of cationic peptides with strong antibacterial activity targeting gram-negative and gram-positive bacteria (Brady et al., 2019). Ballweber et al. found that the antimicrobial peptides D2A21 and D4E1 can maintain anti-Chlamydial activity at a proper concentration in human blood (Ballweber et al., 2002). However, pH values above and below seven reduced D2A21 activity, while the activity of the 2% D2A21 gel formulation remained unchanged at different pH values in their experiment (Ballweber et al., 2002). Whether these gel excipients can make D2A21 peptides exert their inherent activity more fully must be further explored. Interestingly, ultrastructural observations showed that exposure of *C. trachomatis* EBs to peptide D2A21 could lead to membrane dissolution or destruction, but the mechanism is unclear (Ballweber et al., 2002).

Transferrin, a multifunctional protein found in many biological secretions such as milk, tears, and saliva, has anti-inflammatory and antibacterial properties (Wang et al., 2019). Lactoferrin (LF), ovotransferrin (ovoTF), and serum transferrin are the most important members of the transferrin family of iron-binding glycoproteins (Beeckman et al., 2007). Lactoferrin and ovoTF can potentially reduce chlamydial infection *in vivo* and *in vitro* (Beeckman et al., 2007; Wang et al., 2019). Likewise, three transferrins have anti-*C. psittaci* activity, including ovoTF, human lactoferrin (hLF), bovine lactoferrin (bLF), and ovoTF, can stop *C. psittaci* from attaching to and entering the cell (Beeckman et al., 2007). In a follow-up study, turkeys were sprayed with ovoTF to prevent respiratory disease caused by *C. psittaci* (Van Droogenbroeck et al., 2008, 2011). The studies found that ovoTF used in farms can (Zhong, 2017) reduce airborne transmission of *C. psittaci*, (Elwell et al., 2016) reduce the severity of infection, (Cossé et al., 2018) prevent respiratory diseases during the first half of the incubation period, and (Núñez-Otero et al., 2021) produce a synergistic effect with antibiotics. Evidently, the anti-Chlamydial effect of transferrin has been proven not only in *C. psittaci* but also in other *Chlamydia* species. Bovine LF (bLF) can inhibit intravaginal *C. trachomatis* infection and reduce the number of inclusions and the overall replication of *C. suis* in McCoy cells with *C. suis*-spiked semen samples (Sessa et al., 2017; Puysseleyr et al., 2021). Taken together, these studies highlight transferrin's potential diversity in antibacterial efficacy and mechanisms. Other peptides, like spider venom peptides and WLBU2, also have anti-Chlamydial effects (Skinner et al., 2010; Lazarev et al., 2011). Although the exact mechanisms by which these peptides confer antibacterial activity have not been clarified, studies suggest that these peptides have tremendous therapeutic potential against chlamydial infection (Yasin et al., 2004; Mwangi et al., 2019).

### Cytokines

During chlamydial infection, large amounts of cytokines are secreted by host cells that regulate host immune and inflammatory responses. It should be noted that proper responses are beneficial to remove *Chlamydia*, inhibit the infection, and reduce the pathological damage. Conversely, inappropriate responses caused by the excessive release of some cytokines can aggravate the infection. Manipulating these key cytokines may be a new strategy worth investigating for treating chlamydial infection.

Although numerous studies have shown that IFN-γ has an anti-Chlamydial function (Leonhardt et al., 2007; Ohman et al., 2011; Virok et al., 2019; Darville, 2021), no IFN-γ drugs target chlamydial infection. *Chlamydia* and *Mycobacterium tuberculosis* (Mtb) are pathogenic intracellular pathogens that cause a Th1-type immune response, and IFN-γ plays a significant role in resistance to *Chlamydia* and Mtb infections (Desvignes et al., 2012). Thus, the use of IFN-γ treating Mtb infections (Condos et al., 1997; Park et al., 2007; Beeckman and Vanrompay, 2009; Gao et al., 2011) may guide the development of anti-Chlamydial drugs. A previous study (Condos et al., 1997) showed that treatment with IFN-γ *via* aerosol administration helped reduce the bacterial burden in the lungs and even diminished cavitary lesions in a proportion of pulmonary tuberculosis patients. It is worth noting that sputum smears were negative during the 4-week intervention but positive 1–5 months after ending treatment. Further studies must determine whether exogenous IFN-γ long-term treatment can target infectious diseases. Also, observing adverse reactions and the tolerability of treatments is necessary for safety evaluation, even if no systemic side effects occur.

The interleukin (IL) family is an effective group of cytokines that helps promote or inhibit chlamydial infection. Studies have shown that macrophages, Jurkat cells, and THP-1 cells infected with *C. trachomatis* exhibit more IL-10 receptors than uninfected cells (Hakimi et al., 2014). Likewise, the secretion of IL-10 increases in the early stages of *C. trachomatis* infection in the male reproductive tract (Sanchez et al., 2019). Azenabor and York induced *C. trachomatis*-infected macrophages to produce IL-10 by increasing intracellular Ca^2+^ levels (Azenabor and York, 2010). In another study, the IL-10 level of patients with chlamydial infections was higher than that of uninfected individuals (Han et al., 2006). In a study on intranasal infection with *C. psittaci*, IL-10^−/−^ mice were found to promote activation and assembly of the NLRP3 inflammasome, promoting apoptosis, and leading to chlamydial clearance (Li Q. et al., 2021). Studies on mice with IL-10 deletion have shown that the loss of this cytokine distorts the anti-Chlamydial immune response, altering the dominant Th1 phenotype, and preventing *Chlamydia*-induced immunopathology (Bua et al., 2019; Sanchez et al., 2019). Bua et al. also found higher IL-10 levels in infertile women (Bua et al., 2019). Therefore, increased IL-10 expression is not only associated with persistent chlamydial infection but may also be associated with complications of chlamydial infection, such as infertility. Although the mechanism of IL-10 action is still not fully understood, it is undeniable that IL-10 may be a new pathway for chlamydial treatment. Relevant KO mice, siRNA/chemical inhibition, or antibody blockade may be used for identifying the exact mechanism of IL-10 in chlamydial infection. Furthermore, attempting to use the corresponding antibody or inhibitor of IL-10 to control chlamydial infection may be beneficial (Xiang et al., 2021).

Cytokines play a critical role in the fight against tumor cells and pathogens. However, many barriers, such as toxicity-related inherent characteristics, including short half-lives in circulation, inherent pleiotropic functions, and off-target effects, have previously blocked the development of cytokines as immunotherapy drugs (Zheng et al., 2022). With the further study of cytokine immunobiology, immune cytokine drugs are being rapidly developed with new protein engineering and synthetic design technologies, and some have entered clinical trials. For instance, structural engineering can overcome some limitations in the type-I IFN family, including adverse side effects and limited efficacy, and help this family have a broader application prospect in antiviral and antiproliferative clinical practice (Jaitin et al., 2006; Brideau-Andersen et al., 2007; Thomas et al., 2011; Levin et al., 2014). Additionally, studies suggest that selective and accurate modifications of cytokines are useful to enhance their target, efficiency, and long-term efficacy. Moreover, reducing their bioactivity or biological function may be considered a novel way to lessen the toxic reaction (Zheng et al., 2022). In brief, many theories and empirical evidence show that cytokines have therapeutic potential against chlamydial infection but still require future work.

### Vaccines

It is widely known that successful vaccination campaigns have effectively prevented life-threatening diseases such as influenza, tetanus, smallpox, and polio (Vashishtha and Kamath, 2016; Pandolfi et al., 2018; Pollard and Bijker, 2021). Without exception, a chlamydia vaccine is also under development and has achieved great expectations thus far (Zhong et al., 2019; Brunham, 2022). Vaccines effectively prevent infectious diseases and play an important role in treating cancer and other diseases (Polaris Observatory Collaborators, 2018; Calabrese, 2021; Chaudhary et al., 2021). For example, a previous study showed that a multivalent vaccine could protect against *C. trachomatis* infection in vaccinated mice, reduce the *C. trachomatis* load in the vagina, and prevent pathological changes in the upper genital tract (Olsen et al., 2015). Unvaccinated mice had substantial oviduct pathology, such as pronounced lymphocyte infiltration in the mesosalpinx and ovarian bursa after *C. trachomatis* infection, compared to the Hirep1-vaccinated mice with no pathological changes. Chlamydial vaccines can suppress infection or slow disease progression by preventing early chlamydial infection from aggravating (Stary et al., 2015; Paes et al., 2016). In addition, vaccines can induce a strong mucosal immune response to suppress *C. trachomatis* genital infection and reduce long-term sequelae (Ganda et al., 2017). In general, receiving a preventive chlamydial vaccine can induce effective immune responses to prevent and control chlamydial infection in uninfected individuals, but it cannot control existing infections or lesions, and it is not suitable for treating patients. Thus, developing therapeutic vaccines that can contribute to removing pathogens and abnormal cells profoundly influences the treatment of chlamydial infection. Although no therapeutic vaccine has been reported for *Chlamydia*, existing therapeutic vaccine research on cancer, rheumatic disease, and some infectious diseases, including AIDS and chronic hepatitis B virus, provides a great deal of theoretical evidence and practical experience for a Chlamydia vaccine (Burke, 1993; Bertoletti and Le Bert, 2018; Calabrese, 2021).

Several HIV therapeutic vaccines are already being tested in clinical trials. These vaccines function primarily by activating host-specific immune responses and could improve the T-cell subset homeostasis, which is not completely recovered in HIV-treated patients (Leal et al., 2017). Progress has also been made on a therapeutic vaccine for the human papillomavirus (HPV). The therapeutic vaccine for this virus can promote histopathological regression and virus clearance in patients, eliciting an increased frequency of T-cell responses, which are critical for clearing chlamydial infection (Hancock et al., 2018; Garbuglia et al., 2020). HIV, HPV, and *C. trachomatis* are known to be sexually transmitted diseases and/or pathogens. From this perspective, a chlamydial therapeutic vaccine can apply the design of HIV and HPV therapeutic vaccines, although further research is recommended (Gray et al., 2009; Sandoz and Rockey, 2010; Hafner and Timms, 2018; Abraham et al., 2019). In addition, each vaccine platform has its own strengths. For instance, the DNA vaccine can induce antigen-specific immunity, has satisfactory safety and stability, and can be manufactured rapidly. The literature shows that the DNA vaccine has great potential for developing a therapeutic HPV vaccine (Cheng et al., 2018). In conclusion, the design of a chlamydial therapeutic vaccine has excellent potential advantages and feasibility and is a promising candidate treatment strategy for chlamydial infectious diseases. Notably, efficacy and security should be actively considered when finding an effective vaccine optimization strategy, such as dominant antigens, adjuvant delivery systems, and vaccination methods.

## Perspective

Most chlamydial species can lead to infectious diseases and complications in humans and animals; *C. trachomatis* is one of the major causes of STIs. Developing timely screening and precise diagnosis is key to controlling the spread of chlamydial infectious diseases. Nevertheless, many obstructions in screening, diagnosis, and treatment cause the prevention and control of chlamydial infections to be sub-optimal. Due to their high sensitivity and specificity, NAATs are the preferred screening techniques for gonorrhea and *Chlamydia* (National Academies of Sciences, Engineering, and Medicine et al., 2021). However, some factors, such as the high cost, specialized personnel, and lengthy analysis, limit NAAT's application in parts of low- and middle-income countries.

However, the rapid growth in paper microfluidic technologies and isothermal amplification of nucleic acids show a new prospect for sensitive nucleic acid detection tests (Magro et al., 2017). Developing cheaper, faster, more convenient, and more precise diagnostic tools is critical to ensuring prompt treatment and reducing health risks and ongoing STI transmission. Effective treatment is beneficial to better cope with the challenge of the continually rising chlamydial infection rate.

Antibiotic therapy is often the primary clinical treatment against chlamydial infection, but antimicrobial resistance should be considered a potential health threat. Many restrictions and difficulties exist in investigating and overcoming the antimicrobial resistance problems related to anti-Chlamydial infection, such as the lack of a standardized *in vitro* assay, and the uncertain relation between the experiment results *in vitro* and clinical outcomes after antibiotic therapy (Cushnie and Lamb, 2011). To reduce the risk of antibiotic resistance and avoid disrupting the commensal flora, we still need to find compounds that have a selective effect against *Chlamydia*. The developmental cycle of *Chlamydia* is unique and corresponds to its regulation of gene expression. Therefore, targeting the developmental cycle of *Chlamydia* and the transcription of chlamydial virulence may be promising pathways for developing highly selective anti-Chlamydial drugs (Núñez-Otero et al., 2021; Seleem et al., 2022). Although it is useful to control and remove chlamydial infections with current drug treatments, it cannot treat irreversible lesions. Thus, it is vital to develop more effective prevention strategies and therapeutic drugs based on the pathogenic mechanisms of *Chlamydia* (Xiang et al., 2021).

The process of discovering new antibacterial compounds for their clinical application is long and arduous; many compounds are still in the preclinical stage. Taken together, it has been proven that some non-antibiotic substances have the developmental potential to inhibit chlamydial infection. At the same time, efficacy and safety assessments always have some limitations. Pharmacokinetic/pharmacodynamic (PK/PD) modeling and simulation is an innovative technique that links PK profiles with the corresponding PD to improve drug development. Collecting and analyzing PK/PD information for designing optimal dosing strategies, evaluating animal models, and planning clinical studies is crucial. Moreover, some studies suggest that *in vitro* PK/PD models can be used to estimate antibiotic breakpoints, which is important in inhibiting the development of antibiotic resistance. For future regulatory guidance, pharmaceutical companies and sponsors should consider PK/PD as critical topics for drug development (Schmidt et al., 2008; Bhavnani and Rex, 2017).

In conclusion, antibacterial agents must provide good PK/PD support data. Other critical issues of drug development, such as the mechanism of action, specificity, and toxic side effects, should be addressed. Finally, we will only comprehend the control and effectiveness of drugs with ongoing clinical trials.

## Author contributions

All authors listed have made a substantial, direct, and intellectual contribution to the work and approved it for publication.

## Funding

This work was supported by the Natural Science Foundation of Hunan Province (Grant No. 2021JJ40455), the Projects of the Hunan Health Committee (Grant No. 202112071537), the National Innovation and Entrepreneurship Training program for college students (Grant No. 202110555102), the Innovation and Entrepreneurship Training program for college students of the University of South China (Grant Nos. X202010555371 and X202010555372), and Graduate Student Research Innovation Project of Hunan Province (Grant No. CX20221007).

## Conflict of interest

The authors declare that the research was conducted in the absence of any commercial or financial relationships that could be construed as a potential conflict of interest.

## Publisher's note

All claims expressed in this article are solely those of the authors and do not necessarily represent those of their affiliated organizations, or those of the publisher, the editors and the reviewers. Any product that may be evaluated in this article, or claim that may be made by its manufacturer, is not guaranteed or endorsed by the publisher.
